# African origin of *Bradyrhizobium* populations nodulating Bambara groundnut (*Vigna subterranea* L. Verdc) in Ghanaian and South African soils

**DOI:** 10.1371/journal.pone.0184943

**Published:** 2017-09-25

**Authors:** Doris K. Puozaa, Sanjay K. Jaiswal, Felix D. Dakora

**Affiliations:** 1 Department of Crop Sciences, Tshwane, University of Technology, Pretoria, South Africa; 2 Department of Chemistry, Tshwane, University of Technology, Arcadia Campus, Pretoria, South Africa; National Cheng Kung University, TAIWAN

## Abstract

Flavonoids secreted by legumes play a major role as signal molecules for attracting compatible rhizobia. The aim of this study was to assess and understand the diversity of microsymbionts nodulating Bambara groundnut (*Vigna subterranea* L. Verdc.) landraces of different seedcoat colours using restriction fragment length polymorphism and phylogenetic analysis. Seedcoat pigmentation of landraces had effect on the diversity of microsymbionts of Bambara groundnut. Even when planted together in one hole, nodulating bradyrhizobia clustered differently. For example, 16S rDNA-RFLP typing of rhizobial samples TUTVSBLM.I, TUTVSCRM.I and TUTVSRDM.I originating respectively from Black, Cream and Red landraces that were co-planted in the same hole at Manga in the Sudano-sahelian savanna, as well as TUTVSCRK.I and TUTVSRDK.I respectively from Cream and Red landraces co-planted at Kpalisogu in the Guinea savanna, revealed different 16S rDNA- RFLP types. Phylogenetic analysis of 16S rDNA, *glnII*, *recA* and *atpD* sequences showed that *Vigna subterranea* was nodulated specifically by a diverse group of *Bradyrhizobium* species (e.g. *Bradyrhizobium vignae*, and a novel group of *Bradyrhizobium* spp.) in soils from Ghana and South Africa. The *recA* gene phylogeny showed incongruency with the other housekeeping genes, indicating the possibility of lateral gene transfer and/or recombination events. The grouping of isolates according to symbiotic gene (*nifH* and *nodD*) phylogenies revealed inter- and intra-specific symbiotic plasmid transfer and different evolutionary history. The results also showed that a cropping history and physico-chemical environment of soils increased bradyrhizobial diversity in Ghana and South Africa.

## Introduction

Bambara groundnut (*Vigna subterranea* L. Verdc.) is the third most important food legume in Africa after groundnut and cowpea, both in consumption and land area under cultivation. The edible grain of this crop is high in protein (20.6%) and carbohydrate (56.5%), and has 6.6% fat and 6.3% fiber, which make it a complete meal [1]. In Africa, it is cultivated by smallholder farmers either as a monoculture, in rotation with cereals, or in mixed culture with cereals, as well as root and tuber crops [2]. This legume is drought-tolerant and thrives well in nutrient-poor soils largely due to its ability to form effective root nodules with compatible soil rhizobia that reduce atmospheric N_2_ to ammonia for bacterial and plant use. This plant-rhizobia interaction is initiated by a mutual recognition of molecular signals released by both symbiotic partners, and is characterized by varying degrees of specificity pre-determined by the nature and profile of seed/root exudates from the legume, as well as nodulation factors from the rhizobia [3–5]. The plant molecules involved in this initiation step are flavonoids [6–8].

Flavonoids and anthocyanins are secondary metabolites found in most plant parts. They occur in high concentrations in legume seeds [9] and determine the colour of seedcoats. These compunds serve as signal molecules for compatible soil rhizobia during nodule formation [10–12], and are therefore major determinants of specificity in the choice of bacterial partner. Different types of flavonoids have been shown to play an important role in the nodulation of soybean, common bean, alfalfa, cowpea, Bambara groundnut and Kersting’s bean [10, 13–17].

As with many legumes, the seedcoat of Bambara groundnut landraces can vary considerably in colour, ranging from black to mottled, to lighter cream pigmentation due largely to the presence of different types and concentrations of flavonoids [18]. Bambara groundnut landraces are also reported to fix varying amounts of N under field and glasshouse conditions [19–23]. Given the importance of seedcoat compounds in nodule formation, the variation in seed colour of Bambara groundnut grain probably plays a critical role in the choice of microsymbiont partner, through attracting different types of native rhizobia with different levels of symbiotic efficiency.

The application of commercial inoculants is an effective and convenient way of introducing high performing rhizobia to the rhizosphere of legumes [24] for improved nodulation, symbiotic N_2_ fixation, and grain yield. Unfortunately, most of these inoculants contain exotic rhizobial strains whose survival in local soils tends to be low under the prevailing harsh environmental conditions. The net result is usually poor performance. There must however be an abundance of well-adapted indigenous rhizobia in African soils capable of nodulating Bambara groundnut and effectively fixing N_2_ since the crop originates from Africa. Indeed, according to Caballero-Mellado and Martinez-Romero [25], the defined sites of origin of legumes tend to coincide with the diversity centers of their specific symbiotic bacteria. Recently, Gronemeyer et al. [26] reported the presence of a diverse *Bradyrhizobium* group responsible for nodulating Bambara groundnut in Angola and Namibia, and suggested that indigenous rhizboial populations in Africa could be exploited for use as inoculants to increase Bambara groundnut production. The objective of this study was to assess the molecular diversity and phylogeny of indigenous rhizobial populations nodulating Bambara groundnut landraces of different seedcoat colours in Ghana and South Africa.

## Materials and methods

### Description of experimental sites

Field experiments were set up in February 2013 at Morwe (latitude 25.153 and longitude 28.961) in the Nkangala district, Mpumalanga Province, South Africa, with permission from the staff of the Department of Agriculture, Rural Development, Land and Enviornmental Affairs (DARDLEA). The experiments in Ghana were set up during the cropping season in July 2013 at three sites in the Guinea savanna and two sites in the Sudano-sahelian savanna. The three sites in the Guinea savanna were located in Northern Region at Savelugu (latitude 9.624722, longitude 0.827778), Gbalahi (latitude 9.433333, longitude 0.766667) and Kpalisogu (latitude 9.405066, longitude 1.002990). Experiments were done in the Guinea savanna through farmers’ permission via the Extension staff of the Ministry of Food and Agriculture, Northern Region, Ghana. The two sites in the Sudano-sahelian savanna were sited at Googo (latitude 10.7545041, longitude 0.4879915) and Manga (latitude 11.017331, longitude 0.264352), both in the Upper East Region of Ghana. Experiments were done in the Sudano-sahelian savanna through farmers’ permission via the Extension staff of the Ministry of Food and Agriculture, Upper East Region, Ghana. Prior to planting, soil samples were collected from field plots of each site for physical and chemical analysis (Table 1).

**Table 1:** Land use, cropping history and soil physical and chemical properties of sites in Ghana and South Africa prior to setting up of experiments.

Soil parameter	Country	Cropping history	Land use	Texture	pH (KCl)	Total N	Org matter		Ca	K	Na	P	Cu	Mn	Fe
						%			mg.kg^-1^						
Kpalisogu	Ghana	Maize, fallow	Farm land	sandy loam	4.7	0.05	0.88	320		58.5	9.2	8	3.90	31.55	1.50
Gbalahi	Ghana	Maize, Maize	Farm land	sandy loam	5.1	0.06	1.09	640		46.8	6.9	7	3.35	24.8	2.35
Manga	Ghana	Maize, Maize	Research field	loamy sand	4.7	0.05	0.83	214		97.5	11.5	23	4.60	17.95	1.40
Googo	Ghana	Maize, Maize	Farm land	sandy loam	4.6	0.08	1.38	534		140.4	16.1	15	8.15	69.85	1.95
Savelugu	Ghana	Cassava, cassava	Farm land	sandy loam	4.9	0.04	0.67	320		54.6	6.9	8	6.25	29.65	0.95
Morwe	South Africa	Fallow, Various crops	Research field	Loamy sand	5.5	0.02	0.96	164		128	11.0	10	0.45	59.37	ND

ND: Not determined

### Type and source of Bambara groundnut germplasm and collection of root nodules

Seeds of six Bambara groundnut landraces with similar growth habits and phenologies but having different seedcoat colours (Black, Red, Cream, Redmottled, Blackmottled and Blackeye) were collected from local farmers and/or from village markets in the Upper West and Northern Regions of Ghana. The surface sterilized seeds of these landraces were either planted single in a hole, or co-planted together in one hole. At flowering, nodule samples (7–10 plants/plot) were collected at 56 days after planting (DAP), dehydrated in vials containing silica gel, and stored at 4°C until use.

### Nodule rhizobia DNA extraction and 16S rDNA-restriction fragment length polymorphism (16S rDNA-RFLP)

The dried nodules from each of the six Bambara groundnut landraces were rehydrated by soaking in sterile distilled water and surface-sterilized, as described by Somasegaran and Hoben [27]. Single nodules were then crushed in a drop of sterile deionised distilled water in sterilised 2-ml microcentrifuge tubes using sterile plastic pestle. Bacterial genomic DNA was extracted directly from the crushed nodules using GenElute bacterial genomic DNA extraction kit (Sigma-Aldrich, USA) according to the manufacturer’s instructions. Polymerase chain reaction (PCR) was carried out to amplify the 16S-rDNA genomic region of the nodule rhizobial DNA in 25 μl reaction volume containing 5× My Taq PCR buffer, 0.5 U Taq polymerase (Bioline, USA), 10pM each of the primers with standard temperature profile (S1 Table) in Thermal cycler (T100, Bio-Rad, USA).

The amplified 16S-rDNA PCR products were digested with four restriction enzymes (namely, HaeIII, MspI, RsaI and HpaII) according to the instructions of the manufacturer (Thermo Scientific, Lithuania). Aliquots of the enzyme-treated PCR-amplified products were checked using electrophoresis on 3% agarose gel stained with ethidium bromide. The lengths of the separated restricted fragments on the gel were calculated using image lab software (Bio Rad, USA) and the fragments scored as (1) for the presence and (0) for the absence of homologous bands. Thereafter, the similarity of the strains tested was evaluated using Jaccard’s similarity coefficient with NTSYSpc 2.1 software [28]. A dendrogram was constructed from the distance matrix using the mean of unweighted pair group method with arithmetic mean algorithm (UPGMA).

### Constrained correspondence analysis

The 16S rDNA–RFLP data were used to correlate with soil environmental and seedcoat colour data under constrained correspondence analysis (CCA). The analysis was done by means of XLSTAT. The combination of soil environmental variables and seedcoat colours was used to describe the most influential variables for rhizobial diversity by conducting an ANOVA permutation test with 1000 permutations in a reduced model (P<0.05).

### Sequencing and phylogenetic analysis of 16S-rDNA, housekeeping (*atpD*, *recA* and *glnII*) and symbiotic (*nifH* and *nodD*) genes

The PCR amplification of housekeeping (*atpD*, *recA* and *glnII*) and symbiotic (*nifH* and *nodD*) genes of the rhizobial genome was done as described above for 16S-rDNA PCR amplification. The primers used and thermal cycle conditions are listed in S1 Table. The PCR-amplified product of 16S rDNA, housekeeping and symbiotic genes was purified by Favour/Prep PCR purification kit (FAVORGEN, Sigma, USA). The purified samples were sequenced (Macrogen, Netherlands), and the quality of all sequences checked using BioEdit 7.0.0 software [29]. NCBI GenBank databases were used to identify closely related species with test strains using the BLASTn program. The sequences were deposited in the (NCBI) GenBank database to get accession numbers (S2 Table). Reference type sequences were selected to align with sequences of the test strains using MUSCLE [30] for the construction of phylogenic tree by MEGA 6.0 program [31]. Phylogenetic trees were generated by kimura 2-parameter method to calculate evolutionary distances, and evolutionary history was inferred using Neighbor-Joining method [32] algorithm with 1000 bootstrap support [33]. Nucleotide information was obtained from conserved, variable, parsimony-informative, and singleton regions using consensus sequences.

## Results

### Diversity of rhizobial DNA on the basis of 16S rDNA-RFLP analysis

**Fig 1:**
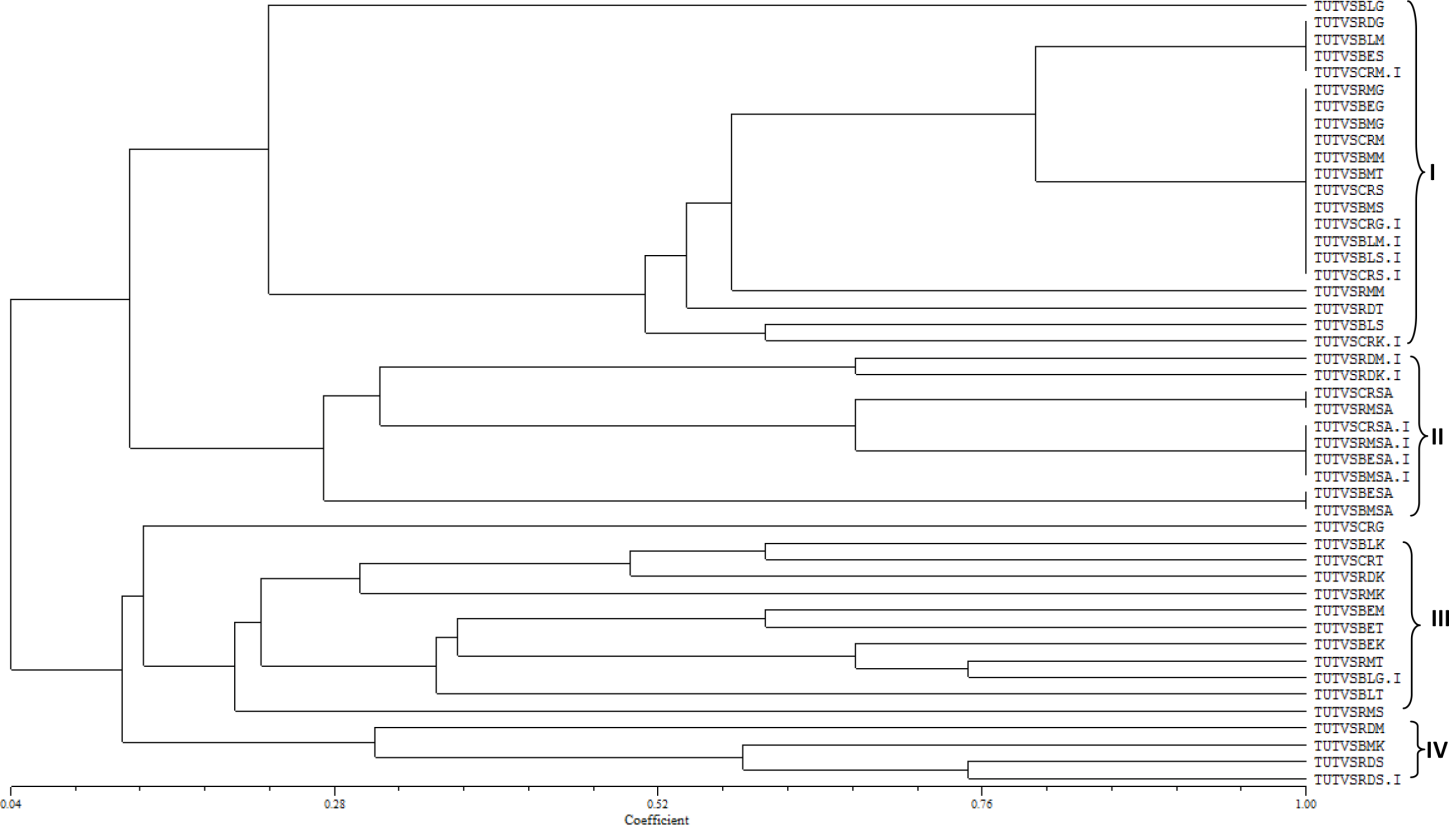
A dendrogram showing the similarities among the PCR-RFLP products of the 16S-rDNA of nodule genomic DNA using four endonucleases (*Hae*III, *Msp*I, *Rsa*I and *Hpa*II).

The PCR-amplified 16S-rDNA genomic region of each of the 47 Bambara groundnut nodule DNA samples tested yielded a single band of approximately 1500 bp. The nodule samples exhibited variable banding patterns of the 16S-rDNA gene when digested with the four restriction endonucleases. The restriction endonucleases produced polymorphic bands, with HaeIII, RsaI and HpaII each yielding six (A-F) banding pattern types, whereas MspI produced eight (A-H; see Table 2). A total of 28 combined 16SrDNA-RFLP types were obtained (Fig 1 and Table 2). A dendrogram generated from the combined banding patterns placed the 47 nodule DNA samples into four clusters with 0.04 to 1.00 Jaccard’s similarity coefficients (Fig 1). From the six Bambara groundnut landraces, a range of four to eight 16SrDNA-RFLP types were obtained for individual genotypes. Cluster I contained 22 nodule samples, while clusters II, III and IV consisted of 10, 12 and four nodule samples, respectively.

**Table 2:** Nodule samples obtained from Bambara groundnut landraces (with different seedcoat colours) at six sites and their 16SrDNA PCR-RFLP pattern types.

Location	Seed-coat colour	Sample designation	16S restriction pattern				Combined 16S RFLP type	Cluster
			*Hae*III	*Msp*I	*Rsa*I	*Hpa*II		
**Googo**	Black	TUTVSBLG	-	B	A	A	1	I
	Cream	TUTVSCRG	A	A	A	B	13	III
	Red	TUTVSRDG	B	B	B	A	2	I
	Red-mottled	TUTVSRMG	B	B	B	C	3	I
	Blackeye	TUTVSBEG	B	B	B	C	3	I
	Black-mottled	TUTVSBMG	B	B	B	C	3	I
	Black	TUTVSBLG.I	A	C	C	A	22	III
	Cream	TUTVSCRG.I	B	B	B	C	3	I
**Manga**	Black	TUTVSBLM	-	B	B	-	2	I
	Cream	TUTVSCRM	B	B	B	C	3	I
	Red	TUTVSRDM	B	C	C	D	25	IV
	Red-mottled	TUTVSRMM	C	B	B	C	4	I
	Blackeye	TUTVSBEM	C	C	C	C	18	III
	Black-mottled	TUTVSBMM	B	B	B	C	3	I
	Black	TUTVSBLM.I	B	B	B	C	3	I
	Cream	TUTVSCRM.I	B	B	-	-	2	I
	Red	TUTVSRDM.I	B	F	D	E	8	II
**Kpalisogu**	Black	TUTVSBLK	D	C	A	B	14	III
	Red	TUTVSRDK	D	C	F	-	16	III
	Red-mottled	TUTVSRMK	E	C	F	-	17	III
	Blackeye	TUTVSBEK	A	C	-	-	20	III
	Black-mottled	TUTVSBMK	F	D	C	-	26	IV
	Black	TUTVSBLK.I	B	G	B	-	6	I
	Cream	TUTVSCRK.I	B	C	B	C	7	I
	Red	TUTVSRDK.I	B	F	D	E	9	II
**Gbalahi**	Black	TUTVSBLT	F	C	-	B	23	III
	Cream	TUTVSCRT	D	C	A	C	15	III
	Red	TUTVSRDT	B	B	C	C	5	I
	Redmottled	TUTVSRMT	A	C	C	B	21	III
	Blackeye	TUTVSBET	C	C	C	B	19	III
	Black-mottled	TUTVSBMT	B	B	B	C	3	I
**Savelugu**	Black	TUTVSBLS	B	E	B	C	6	I
	Cream	TUTVSCRS	B	B	B	C	3	I
	Red	TUTVSRDS	A	D	C	D	27	IV
	Red-mottled	TUTVSRMS	C	C	B	A	24	III
	Blackeye	TUTVSBES	B	B	B	A	2	I
	Black-mottled	TUTVSBMS	B	B	B	C	3	I
	Black	TUTVSBLS.I	B	B	B	C	3	I
	Cream	TUTVSCRS.I	B	B	B	C	3	I
	Red	TUTVSRDS.I	A	D	C	D	28	IV
**Morwe**	Cream	TUTVSCRSA	B	G	D	F	10	II
	Red-mottled	TUTVSRMSA	B	G	D	F	10	II
	Blackeye	TUTVSBESA	B	H	E	F	12	II
	Black-mottled	TUTVSBMSA	B	H	E	F	12	II
	Cream	TUTVSCRSA.I	B	G	D	E	11	II
	Red-mottled	TUTVSRMSA.I	B	G	D	E	11	II
	Blackeye	TUTVSBESA.I	B	G	D	E	11	II
	Black-mottled	TUTVSBMSA.I	B	G	D	E	11	II

The Black and Red landraces each had eight 16S-rDNA types in different clusters of the dendrogram, while landraces Blackeye, Redmottled and Cream each comprised seven 16S rDNA-RFLP types. Nodule DNA samples TUTVSBLG.I and TUTVSCRG.I originating respectively from Black and Cream landraces but co-planted in the same hole belonged to different 16S rDNA-RFLP types, as well as to different major clusters. Similarly, isolates TUTVSBLM.I, TUTVSCRM.I and TUTVSRDM.I respectively obtained from nodule DNA of Black, Cream and Red landraces co-planted in the same hole at Manga in the Sudano-sahelian savanna each showed a different RFLP type. Also, intra-hole DNA samples TUTVSCRK.I and TUTVSRDK.I obtained respectively from genotypes Cream and Red landraces planted at Kpalisogu in the Guinea savanna revealed different 16S rDNA-RFLP types.

The Bambara groundnut nodule samples from Gbalahi and Googo sites in the Guinea savanna respectively comprised six and five 16S rDNA-RFLP types that were distributed in major Cluster I and III. The majority of the nodule DNA samples from Manga in the Sudano-sahelian savanna were grouped in Cluster I. Furthermore, seven 16S rDNA-RFLP types were found in nodule samples from Kpalisogu and were distributed among all four clusters. At Savelugu, a total of nine nodule isolates were analysed and four of them were grouped as 16S rDNA- RFLP type 3, while each of the other five belonged to a different RFLP type. Contrary to the distribution pattern observed in the DNA samples from Ghana, all the bacterial DNA isolates from Morwe in South Africa, were grouped in Cluster II. Three distinct 16S rDNA-RFLP types were revealed. Furthermore, DNA isolates TUTVSCRSA.I, TUTVSBESA.I, TUTVSBMSA.I and TUTVSRMSA.I which respectively originated from nodules of landraces with Cream, Blackeye, Blackmottled and Redmottled seedcoats, when planted in the same hole, grouped together as RFLP type 11, an indication that they were nodulated by very closely related microsymbionts.

### Constrained correspondence analysis

**Fig 2:**
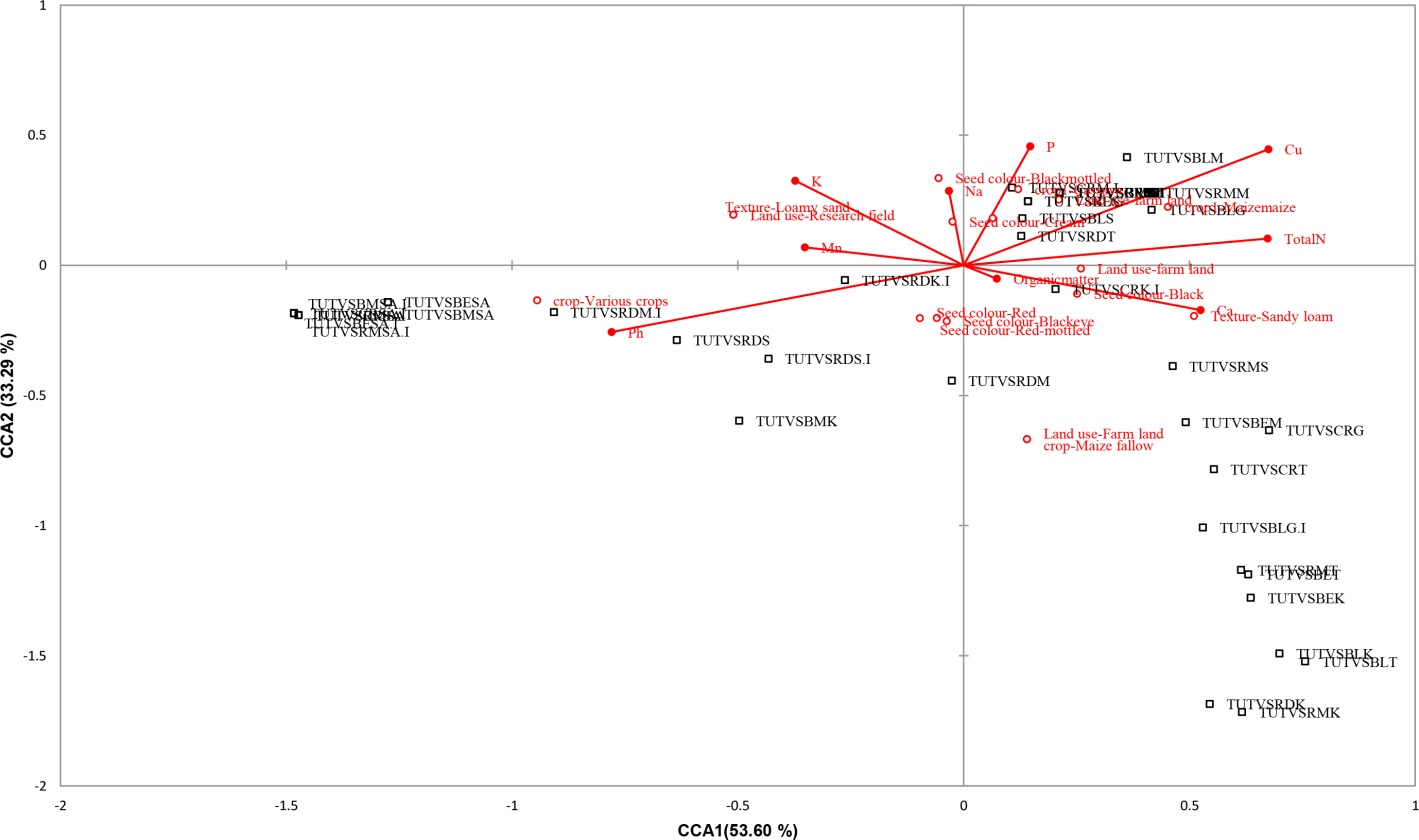
CCA co-ordination plot for the correlation between Bambara groundnut rhizobial communities and environmental variables across locations.

Permutation test showed that the sites/bradyrhizobial data were linearly and significantly (*p≤*0.05) related to the site/environmental data. The constructed CCA plot explained the differences in a set of bradyrhizobial species through variation in a set of environmental variables measured in the same location. The CCA analysis showed that most of the environmental variables were positively correlated with rhizobial diversity. A total of 37.75% constrained inertia was obtained. The constrained axes showed 86.89% linear combination of the environmental variables. The level of constrained inertia expressed by CCA1 and CCA2 was 53.60 and 33.29%, respectively. The CCA triplot showed that most of the isolates, including TUTVSBLM, TUTVSRMM, TUTVSBLG, TUTVSBLS, TUTVSRDT, TUTVSRMS, TUTVSBEM, TUTVSCRG, TUTVSCRT, TUTVSBLG.I, TUTVSRMT, TUTVSBET, TUTVSBEK, TUTVSBLK and TUTVSBLT, were more likely to be found in fields where the previous crops were maize and casava in environments with sandy loam soil texture, while all the South African isolates along with isolates TUTVSRDM.I, TUTVSRDS, TUTVSRDS.I and TUTVSBMK were likely to be found in fields previously grown with various crops. The high soil Cu, followed by total N, Ca, P, K, as well as organic matter environments supported growth of most isolates from Ghana, while high pH and low soil nutrients were noted for South African isolates. Bambara seedcoat colour (Black, Blackmottled and Cream) also provided an environment for rhizobial richness in soil (Fig 2).

### Phylogenetic analysis of 16S-rDNA sequences

**Fig 3:**
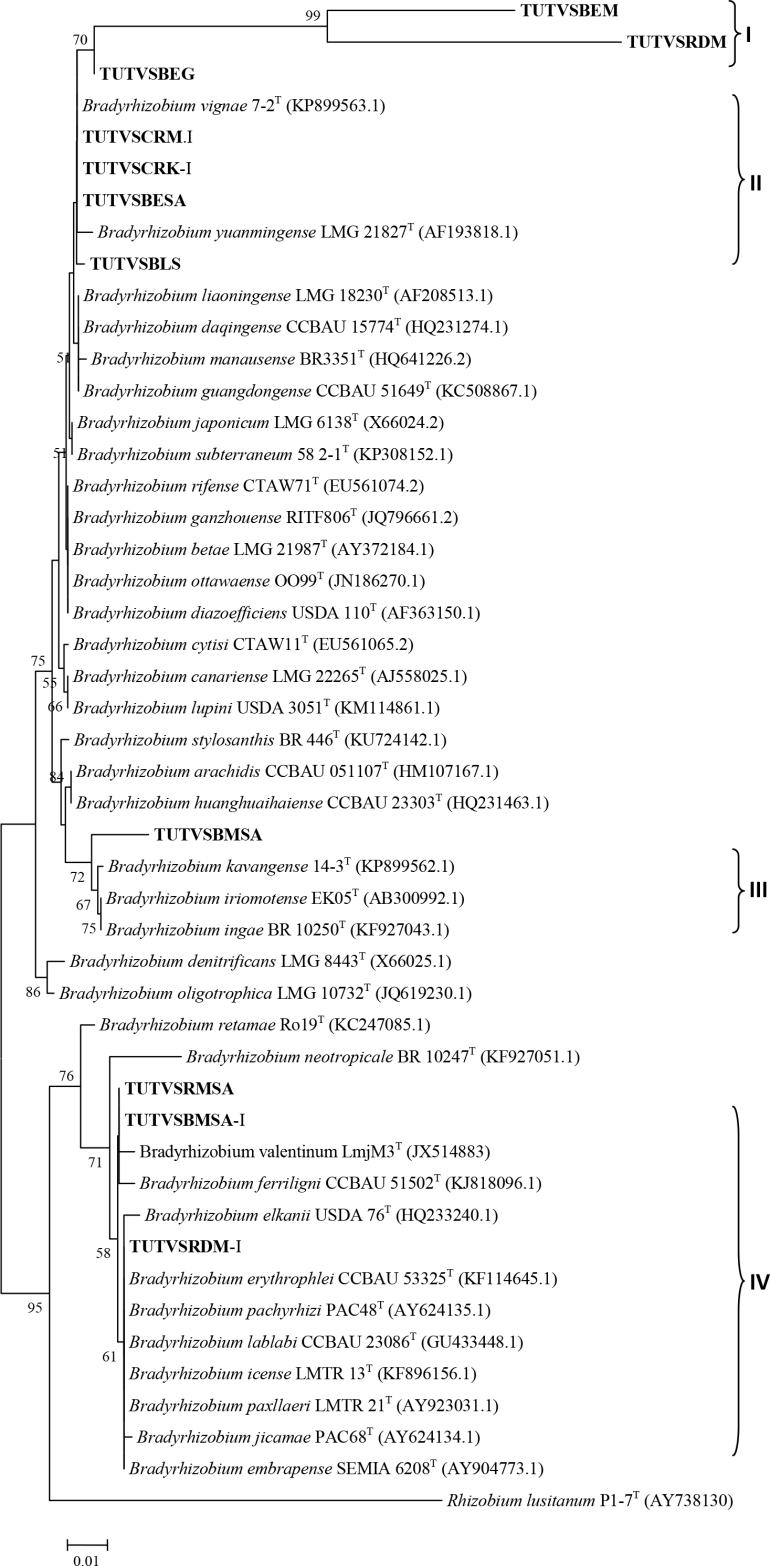
Phylogenetic relationships of microsymbionts from Bambara groundnut root nodules and reference type strains based on 16S-rDNA gene analysis. Phylogeny was inferred using the neighbor-joining method. The percentages of replicate trees in which the associated taxa clustered together were obtained using bootstrap test with 1000 replications.

Representative nodule DNA samples selected from each cluster of 16S-rDNA RFLP were used for phylogenetic analysis. The 16S-rDNA sequences of the test samples showed varying similarities with diverse *Bradyrhizobium* species. The 16S-rDNA tree grouped the nodule DNA samples into four distinct clusters (Cluster I-IV; see Fig 3). Nodule DNA samples TUTVSBEM and TUTVSRDM isolated from Black eye and Red Bambara groundnut landraces that were grown at Manga in the Sudano-sahelian zone grouped together in Cluster I with 99% bootstrap support, but without any reference *Bradyrhizobium* strain. In Cluster II, nodule samples collected from Black, Black eye and Cream Bambara groundnut grown at Manga (Sudano-sahelian zone in Ghana) and Morwe (savanna in South Africa) were closely related in a similar manner as observed in the dendrogram of the combined 16S rDNA-RFLP fingerprints, and grouped with *Bradyrhizobium vignae* with 99.8–100% sequence identity. Nodule sample TUTVSBMSA proximally related to *Bradyrhizobium kavangense* with 72 bootstrap support and 98.2% sequence identity. However, nodules of Red (TUTVSRDM-I), Red mottled (TUTVSRMSA) and Black mottled (TUTVSBMSA.I) in Cluster IV aligned with *Bradyrhizobium elkanii* species with 71 bootstrap support and 98.8–100% sequence identity.

### Phylogenetic analysis of housekeeping gene (*atpD*, *glnII* and *recA*) sequences

The phylogenetic relationship of *atpD*, *glnII* and *recA* genes, which respectively code for ATP synthase beta chain, glutamine synthetase II, and recombinase A, was determined. The PCR amplification of the three housekeeping genes resulted in single monomorphic fragments with band lengths of 550 bp for *atpD*, 500 bp for *glnII* and 600 bp for *recA*.

The BLAST_n_ analysis of nodule bacterial housekeeping gene sequences showed that most nodule occupants belonged predominantly to the *Bradyrhizobium* genus. These protein-encoding nucleotide sequences showed variable informative positions (S3 Table). Several housekeeping gene sequences obtained from the GenBank were shorter than those of test isolate sequences. As a result, after the final alignments, hanging test sequences were omitted, and only the shorter lengths of the housekeeping gene nucleotide sequences of test isolates were used for phylogenetic analysis. The lowest (53.32%) level of conserved and the highest (40.36%) level of variable sequences were observed for *recA*, while *atpD* and *glnII* recorded 64.46% and 63.16% conserved sequences, respectively (S3 Table).

**Fig 4:**
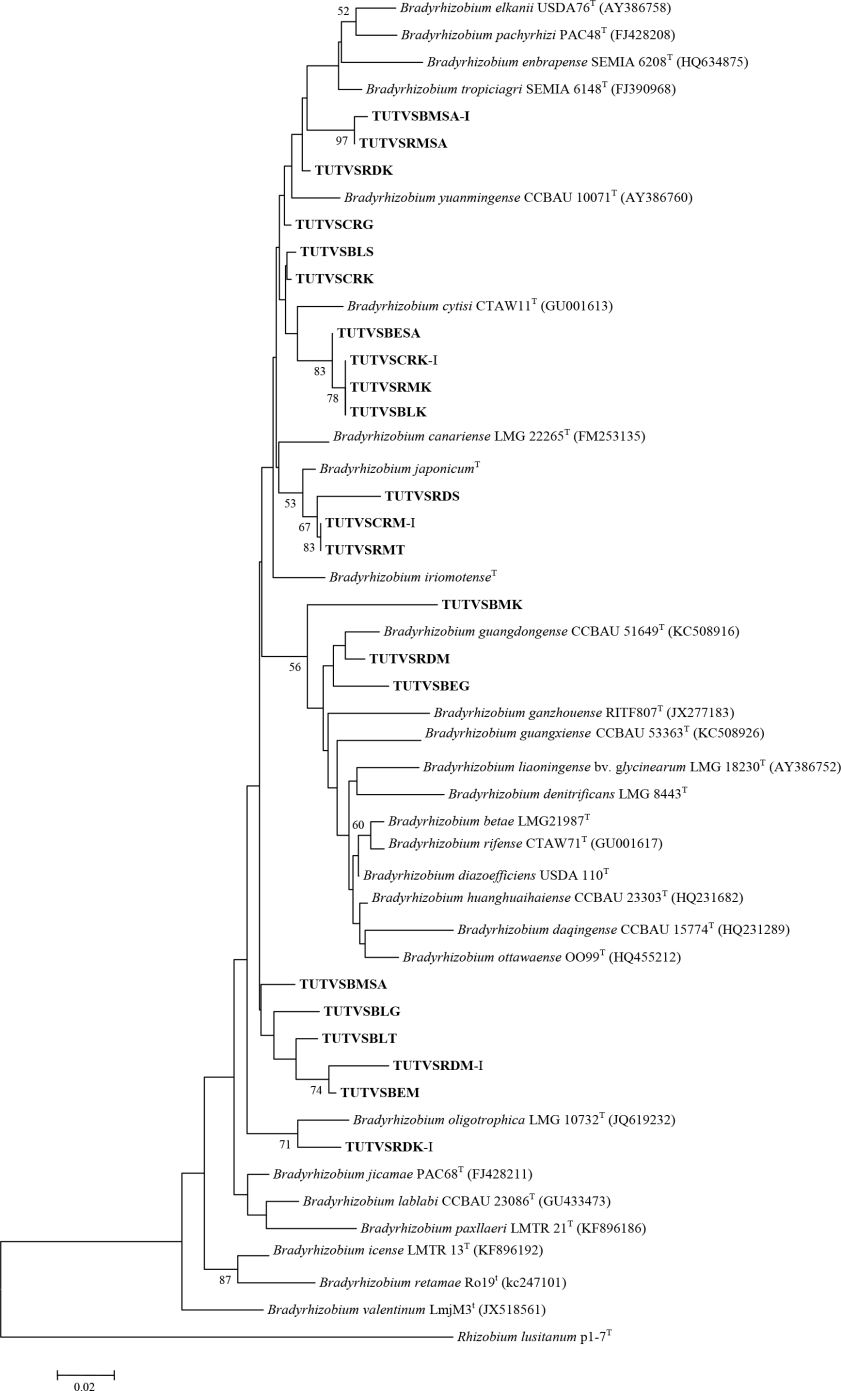
Phylogenetic relationships of microsymbionts from Bambara groundnut root nodules and reference type strains based on *atpD* analysis. Phylogeny was inferred using the neighbor-joining method. The percentages of replicate trees in which the associated taxa clustered together were obtained using bootstrap test with 1000 replications.

**Fig 5:**
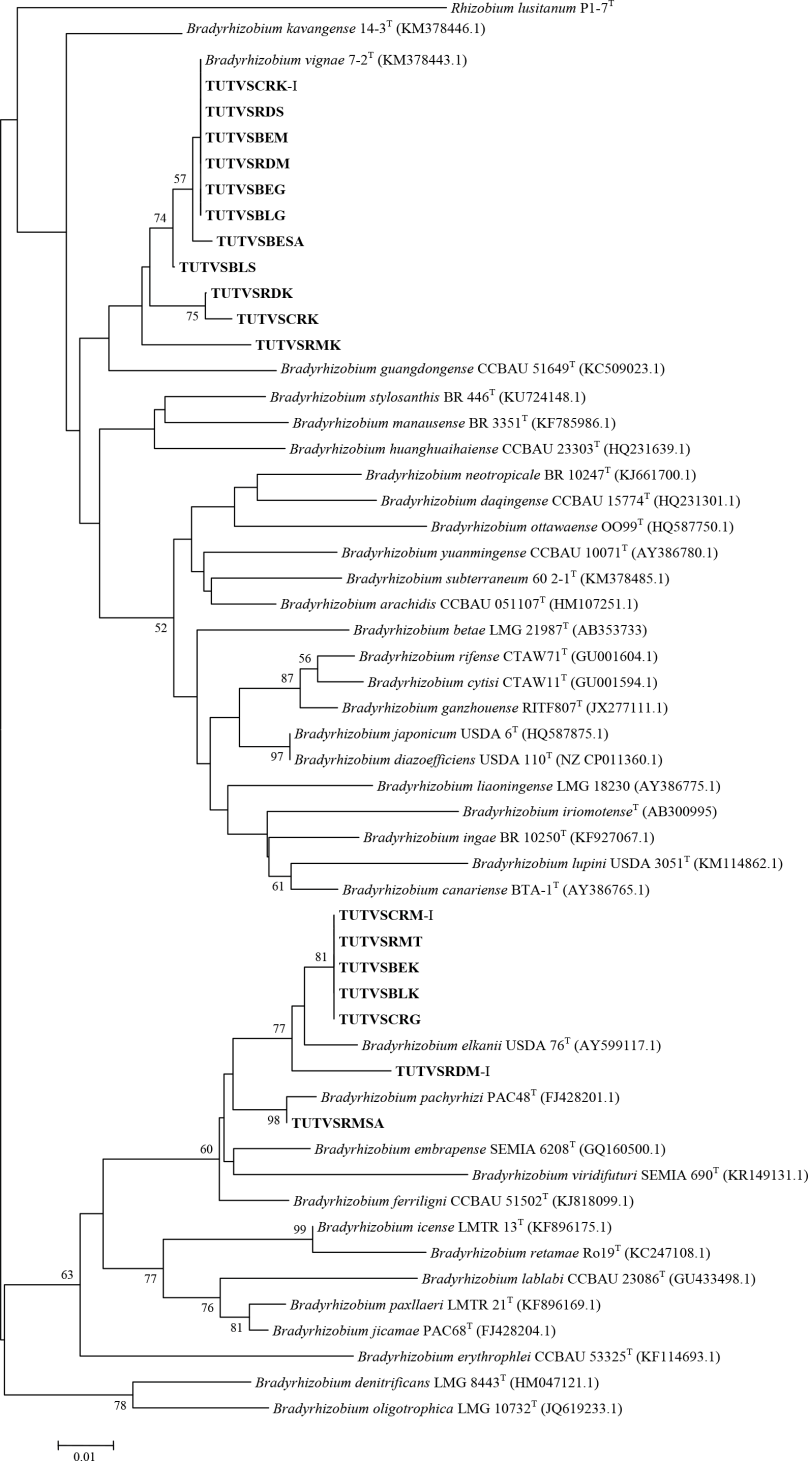
Phylogenetic relationships of microsymbionts from Bambara groundnut root nodules and reference type strains based on *glnII* gene analysis. Phylogeny was inferred using the neighbor-joining method. The percentages of replicate trees in which the associated taxa clustered together were obtained using bootstrap test with 1000 replications.

**Fig 6:**
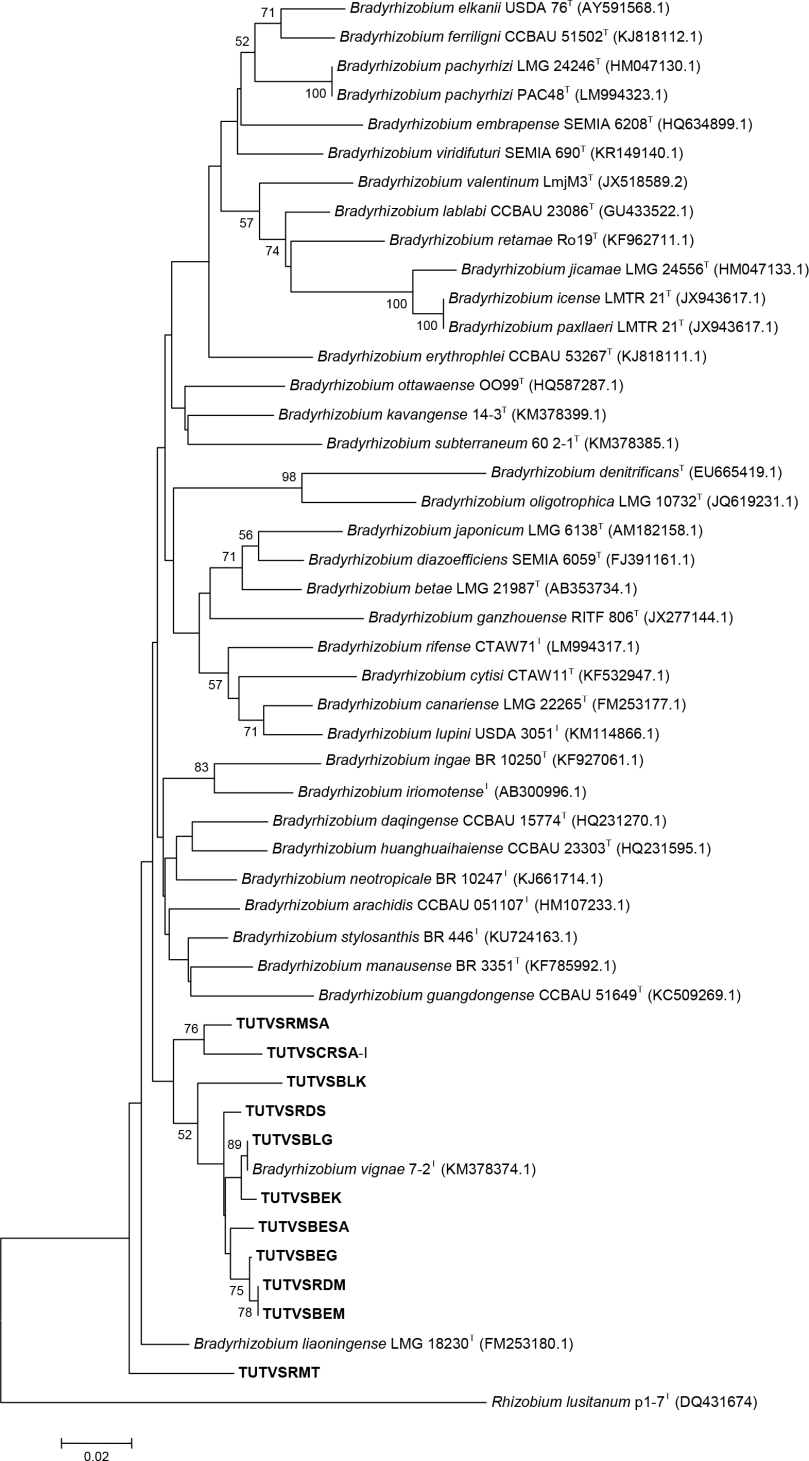
Phylogenetic relationships of microsymbionts from Bambara groundnut root nodules and reference type strains based on *recA*. Phylogeny was constructed using the neighbor-joining method. The percentages of replicate trees in which the associated taxa clustered together were obtained using bootstrap test with 1000 replications.

Phylogenetic trees of all test housekeeping genes were constructed using the neighbor joining method, and this resulted in the grouping of test isolates with different reference *Bradyrhizobium* species (Figs 4–6). Some test sequences were excluded in tree construction either due to incompatibility of the primer pairs used in PCR amplification, or poor sequence quality. In *glnII* phylogram, most of the DNA isolates grouped into two major clusters with *B*. *vignae* and *Bradyrhizobium elkanii*, while isolates TUTBESA, TUTVSBLS, TUTVSRDK, TUTVSCRK and TUTVSRMK were an outgroup of *B*. *vignae* (Fig 5).

In the phylogenetic tree of *glnII* and *recA* housekeeping genes, some of the test DNA isolates (e.g. TUTVSRDS, TUTVSBLG, TUTVSBESA, TUTVSBEG, TUTVSRDM and TUTVSBEM) consistently grouped into a major clade with *B*. *vignae*. Due to the absence of *atpD* sequence of *B*. *vignae* in the GenBank, this reference strain was not included in the *atpD* tree for a clearer position of its grouping in the tree. However, major shifts of the isolates were observed between clusters for grouping with reference type strains in all test housekeeping gene phylogenies. Single gene phylogenetic analysis showed incongruency with some isolates grouping with type strains in all studied phylograms. Isolates TUTVSRMSA, TUTVSBLK, TUTVSBEK and TUTVSRMT grouped with *Bradyrhizobium elkanii* in the *glnII* phylogeny but aligned with *B*. *vignae* in *recA* phylogram, while in the *atpD* phylogeny they aligned with different *Bradyrhizobium* species (Figs 4–6). There was no seedcoat colour-based grouping of isolates with type strains in these single gene phylogenetic analyses.

### Phylogenetic analysis of combined *atpD*, *glnII* and *recA* gene sequences

**Fig 7:**
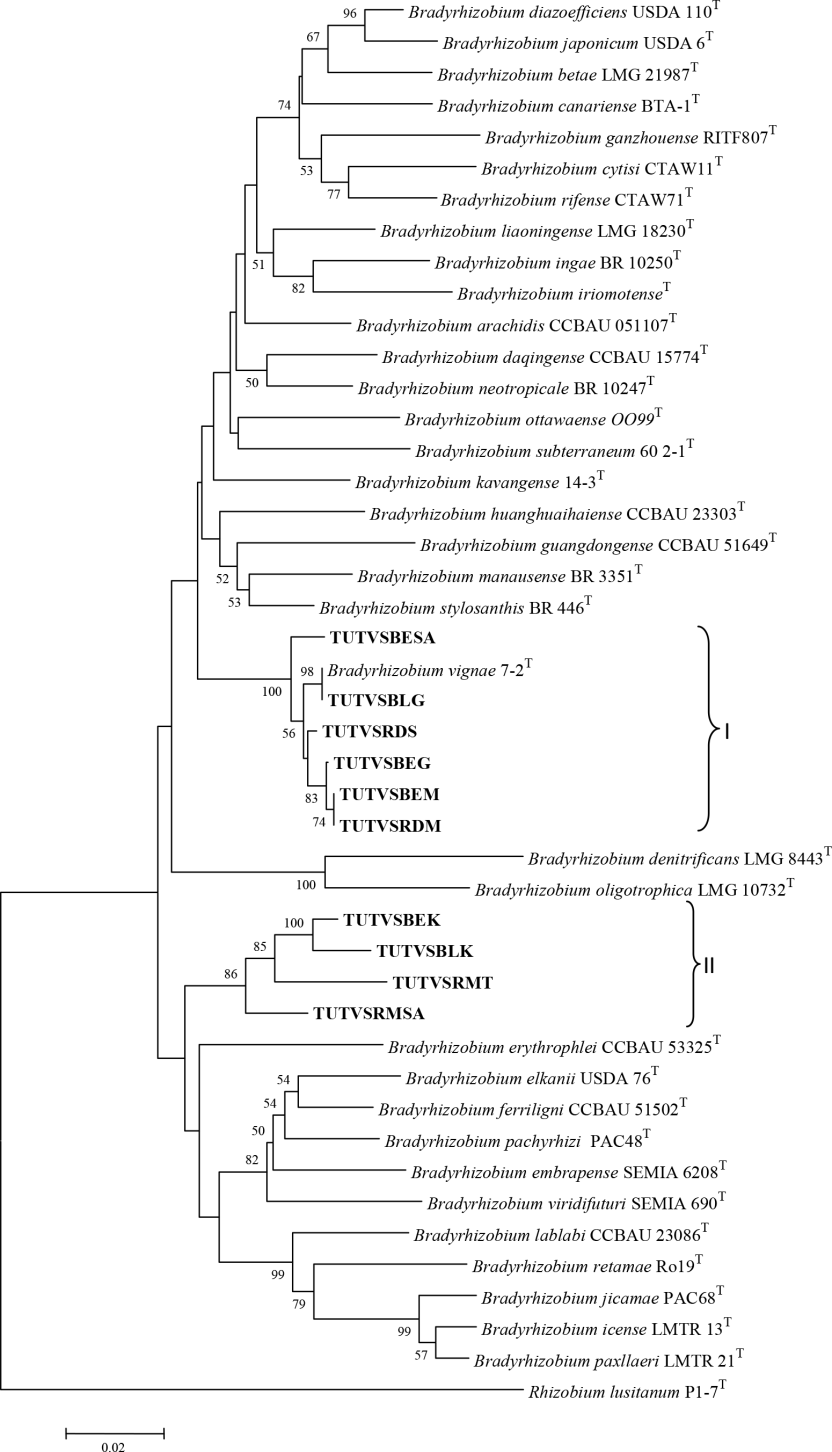
Phylogenetic tree based on *recA*+*glnII* sequences generated by neighbor-joining method. Bootstrap values (1000 replicates) are indicated above the branches.

The concatenated housekeeping gene sequence analysis showed a clearer view of isolate identity and confirmed the presence of diverse and novel type of *Bradyrhizobium* species nodulating Bambara groundnut in Ghana and South Africa. Due to the absence of *atpD* sequence of *B*. *vignae*, two concatenated trees (*recA+glnII*+*atpD* and *recA+glnII*) were constructed to unravel the delineation of nodule samples with known type strains. A phylogenetic tree based on combined *recA+glnII* sequences confirmed the close relationship of these isolates. The constructed concatenated tree placed the nodule samples in two clusters (Cluster I and II). The clustering of DNA isolates was the same in the two concatenated trees (*recA+glnII*+*atpD* and *recA+glnI)*. Nodule isolates TUTVSBESA, TUTVSBLG, TUTVSRDS, TUTVSBEG, TUTVSBEM and TUTVSRDM in Cluster I aligned with *B*. *vignae* with 100% bootstrap support and 98.8–100% sequence identity in the *recA+glnII* concatenated phylogram, but formed a separate cluster in *recA+glnII+aptD* concatenated tree due to the absence of *B*. *vignae*. But nodule DNA samples TUTVSBEK, TUTVSBLK, TUTVSRMT and TUTVSRMSA grouped together in Cluster II without any reference type strains (Fig 7).

### Phylogenetic analysis of *nifH* and *nodD* gene sequences

**Fig 8:**
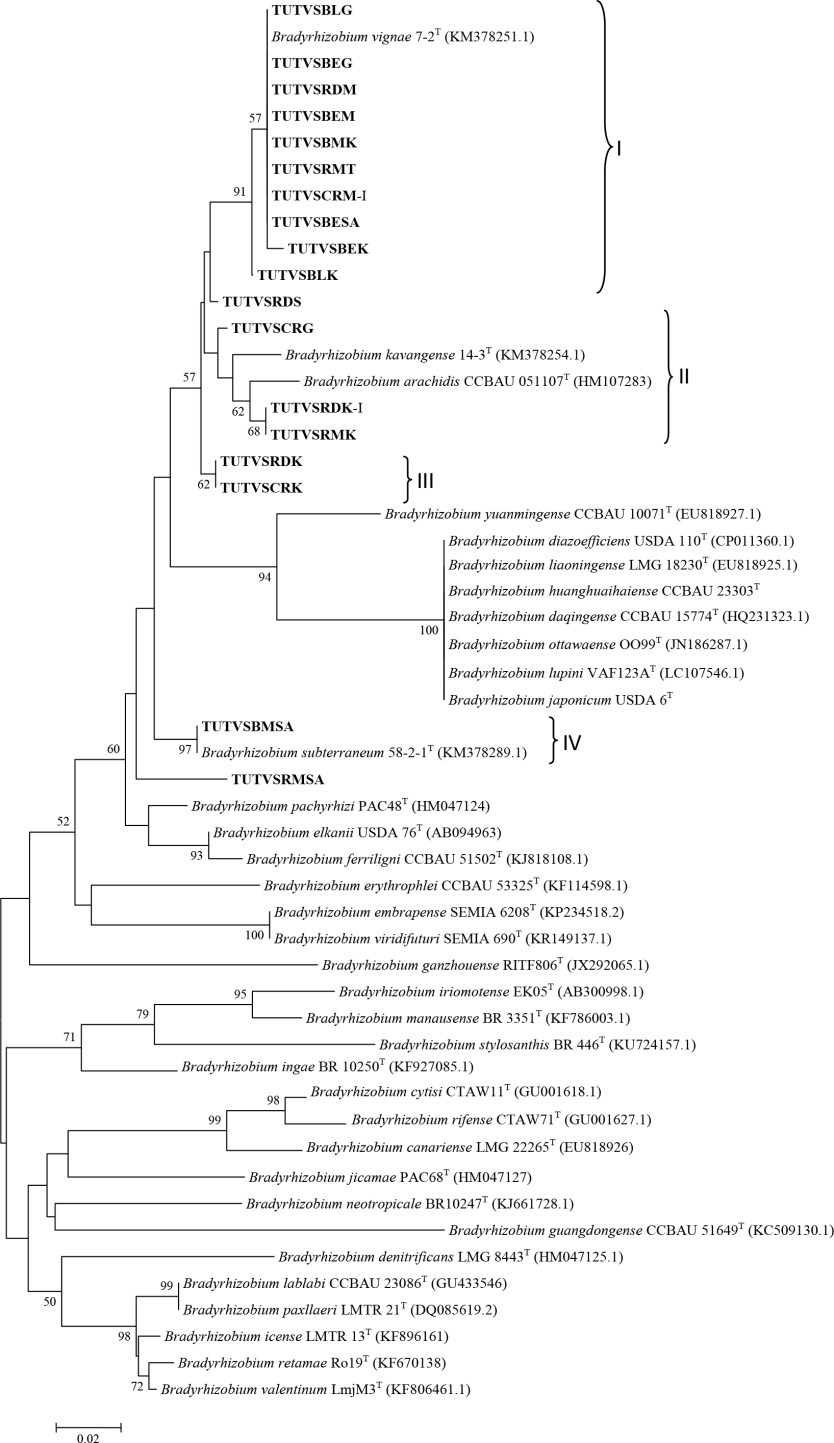
Phylogenetic relationships of microsymbionts from Bambara groundnut root nodules and reference type strains based on *nifH*. Phylogeny was inferred using the neighbor-joining method. The percentages of replicate trees in which the associated taxa clustered together were obtained using bootstrap test with 1000 replications.

PCR-amplification of nodulation and N_2_ fixation genes *nodD* and *nif*H genomic regions yielded single monomorphic bands of about 300 bp and 620 bp, respectively. The *nif*H phylogenetic analysis placed the nodule bacterial DNA samples into four major groups (Cluster I-IV). Most of the nodule bacterial samples were grouped with *B*. *vignae* with 100% sequence identity in Cluster I. TUTVSBLK and TUTVSRDS were however an outgroup of Cluster I with 98–99.5% sequence identity with *B*. *vignae*. Nodule isolates TUTVSCRG, TUTVSRDK-I and TUTVSRMK in Cluster II were proximally related to *Bradyrhizobium arachidis* with 98.5% sequence identity, while TUTVSRDK and TUTVSCRK in Cluster III grouped together without any reference type strain with 100% sequence identity. Single nodule isolate TUTVSBMSA grouped with *Bradyrhizobium subterraneum* with 100% sequence identity and 97% bootstrap support, while TUTVSRMSA stood alone as an outgroup (Fig 8).

**Fig 9:**
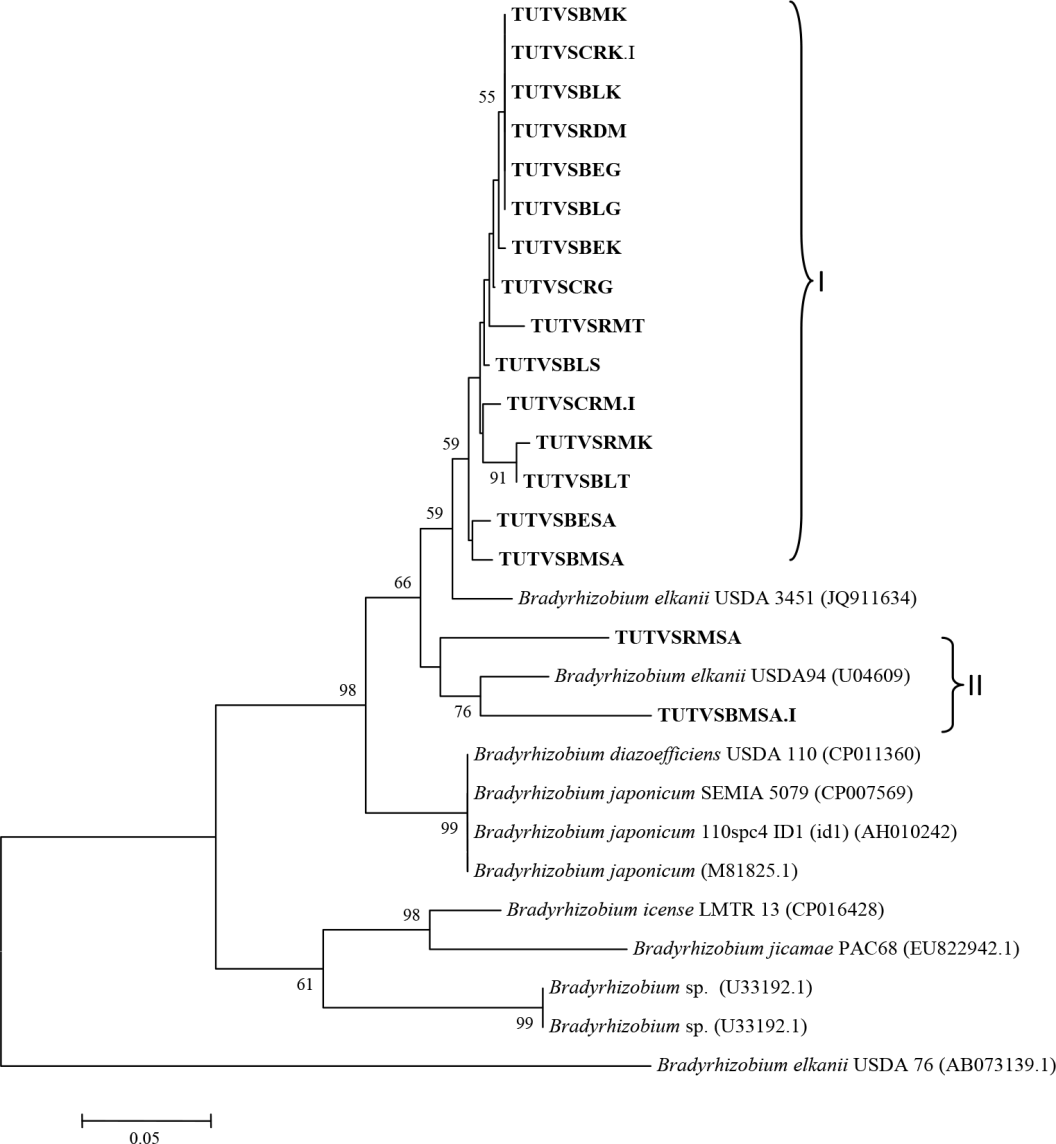
Phylogenetic relationships of microsymbionts from Bambara groundnut root nodules and reference type strains based on *nodD*. Phylogeny was inferred using the neighbor-joining method. The percentages of replicate trees in which the associated taxa clustered together were obtained using bootstrap test with 1000 replications.

The nodulation gene (*nodD)* phylogenetic analysis placed the nodule DNA samples in two clusters, with most of the test samples grouping together in Cluster I without any reference type strain. However, TUTVSRMSA and TUTVSBMSA.I from South Africa were proximally related to *B*. *elkanii* USDA94 (Fig 9).

## Discussion

### Molecular diversity of Bambara groundnut-nodulating bradyrhizobia based on 16S rDNA-RFLP analysis

Bambara groundnut is cultivated across the entire African continent, from Mauritania and Senegal in the West to Uganda and Tanzania in the East, and from Mali and Sudan in the North to South Africa and Zimbabwe in the South. It is therefore important that studies of its microsymbiont diversity covers different agro-ecologies. This justifies the choice of study sites in the contrasting Guinea and sahelian savannas of Ghana, and another savanna in South Africa, where the legume is commonly grown as a food crop. A recent study has shown that Bambara groundnut is nodulated by a diverse group of *Bradyrhizobium* species in Angola and Namibia [26]. In this study, we further explored the diversity of Bambara groundnut-nodulating bacteria using African landraces of different seedcoat colours planted in three distinctly different agro-ecologies (namely, the Guinea and Sudano-sahelian savanna of Ghana, and a savanna in South Africa).

About 47 genomic DNA samples were isolated from root nodules of Bambara groundnut with different seedcoat colour and molecularly characterised. The 16SrDNA-RFLP cluster analysis of the combined restriction patterns from four endonucleases (HaeIII, MspI, RsaI and HpaI) grouped the 47 DNA samples into 28 unique banding patterns in four major clusters (Fig 1). Sample TUTVSCRG, which was extracted from root nodules of the Cream Bambara groundnut landrace planted at Googo in the Guinea savanna of Ghana, did not belong to any cluster, thus indicating very low similarity with the rest of the 46 genomic DNA samples. However, the DNA isolates from root nodules of the five landraces planted at Morwe in South Africa grouped together in Cluster II. This variation in nodule occupancy of Bambara groundnut is consistent with the observed differences in diversity of indigenous bradyrhizobia nodulating Bambara groundnut in Namibian and Angolan soils (26).

The experimental sites in Ghana had a cropping history of maize and cassava cultivation under a monoculture system (Table 1). As a result, the rhizobial populations from these sites were highly diverse, as shown by the clustering pattern (Figs 1 and 2). This observation is consistent with the reportedly high rhizobial diversity associated with groundnut cultivated after a wheat and cocoa monoculture [34–35]. By contrast, the experimental field at Morwe in South Africa was previously fallowed and later intercropped to different food plant species, a land-use system that appeared to have restricted the genetic diversity of bradyrhizobia nodulating Bambara groundnut at that site. In fact, Ngo Nkot *et al*. [35] reported similar findings for rhizobia collected from fields under mixed culture in Cameroon. Cropping systems therefore seem to have a critical influence on the composition of soil microbial populations. But differences in soil fertility (Table 1) also showed visible effect on the distribution of native rhizobia (Fig 2).

Besides cropping systems, seedcoat pigmentation also had an effect on the diversity of microsymbionts isolated from Bambara groundnut root nodules. We found that, even when planted in one hole, landraces of different seedcoat colours nodulated with bacterial partners that clustered differently at the 16S rDNA-RFLP level, except for the isolates from South Africa in Cluster II. A clearer insight was made from the 16S rDNA-RFLP typing of nodule DNA samples TUTVSCRK.I and TUTVSRDK.I obtained from Cream and Red Bambara groundnut landraces that were planted in one hole. Although they shared the same rhizosphere niche, they attracted different rhizobial genotypes, as shown by the different 16S rDNA -RFLP patterns (Table 2). It has been suggested that legumes play a critical role in enriching the soil environment with *Rhizobium* populations through rhizosphere effects [36]. According to Venkateswarlu *et al*. [37] and Hartmann *et al*. [38], the presence of a particular legume in a soil selects for specific rhizobial groups as a result of rhizodepositions which emanate from the host plant. With most legumes, these rhizodepositions are flavonoids [18, 39–40], which occur abundantly in the seedcoat [9]. In fact, legume genotypes with different seedcoat colours generally contain different flavonoid profiles. For example, Bambara groundnut landraces with different seedcoat pigmentation were found to contain different levels of flavonoids and anthocyanins in seed exudates [18]. These seedcoat phenolic compounds were found to serve as chemo-attractants for rhizobia [10], bacterial growth promoters [8] and/or *nod* gene inducers in alfalfa and common bean [13–14, 41–42]. Thus, the different seedcoat compounds of the test Bambara groundnut landraces could also have attracted different rhizobial strains to the rhizosphere. However, the lack of congruence in the clustering of the rhizobial strains from each Bambara groundnut genotype with a different seedcoat colour could imply the existence of different profiles and concentrations of flavonoids even from seeds of the same seedcoat pigmentation.

### Phylogeny of microsymbionts nodulating Bambara groundnut landraces based on multilocus sequence analysis (MLSA)

MLSA has been used extensively in the study of legume rhizobia and is now recognized as a more accessible and affordable tool for phylogenetic and taxonomic studies of root-nodule bacteria when compared to DNA-DNA hybridization [43]. Based on the phylogenetic analysis of the 16S rDNA gene sequences, the bacteria nodulating Bambara groundnut in this study were closely related to *Bradyrhizobium*, and grouped into four clusters with diverse *Bradyrhizobium* species. For clearer delineation, the sequencing of more than one gene has been recommended for taxonomic inferences [44]. Consistent with the results obtained with the 16S rDNA gene sequence analysis in this study, the sequence data of *nifH* and three housekeeping genes (*atpD*, *recA* and *glnII*) of the nodule occupants of the five Bambara groundnut landraces also aligned with diverse species of *Bradyrhizobium*. Single gene phylogenetic analysis showed that some of the nodule bacteria in this study were related to *Bradyrhizobium vignae*, which nodulates *Vigna subterranea*, *Vigna unguiculata*, *Arachis hypogaea* and *Lablab purpureus* [45]. In the phylogenetic trees constructed from sequences of *atpD*, *recA* and *glnII* genes, some monophyletic groups were identified which showed no close relationship with any reference strains. These test sequences probably constitute a novel group of *Bradyrhizobium* yet to be described. Due to the absence *atpD* nucleotide sequence of *B*. *vignae* in the GenBank, we constructed two concatenated trees based on *recA + glnII* and *recA+glnII+atpD*. Both concatenated phylogenies showed similar and congruent topologies.

The phylogenetic analysis of housekeeping genes suggested that some South African and Ghanaian nodule isolates (e.g. TUTBESA, TUTVSBLG, TUTVSRDS, TUTVSBEG, TUTVSBEM and TUTVSRDM) belong to *B*. *vignae* isolated from soils in Angola and Namibia with 100 bootstrap support and 98.8–100% sequence identity. However, the phylogenetic positions of nodule DNA isolates TUTVSRDK, TUTVSCRK, TUTVSRMSA, TUTVSBLK, TUTVSBEK, TUTVSCRG, TUTVSRMT, TUTVSCRM-I and TUTVSBLK-I were not stable in single gene analysis, and grouped with different *Bradyrhizobium* species, while with combined gene analysis, they grouped together separately without any reference type strains. So far, only *Bradyrhizobium* species of African origin are reported to nodulate *Vigna subterraenea*, a finding consistent *with* the report by Caballero-Mellado and Martinez-Romero [25] that the centres of origin of legumes tend to coincide with the diversity centres of their specific bacterial symbionts.

Since there was no history of inoculation at any of the experimental sites used in this study, the microsymbionts present in root nodules of the five Bambara groundnut landraces were considered to be truly authentic indigenous occupants. The results of this study indicate the predominance of unique *Bradyrhizobium* species nodulating Bambara groundnut landraces in Ghana and South Africa. However, it appears previous land-use systems can significantly alter the diversity of bradyrhizobia nodulating Bambara groundnut.

Nodule DNA isolates TUTVSRMSA, TUTVSBLK, TUTVSBEK and TUTVSRMT in *glnII* phylogram were closely related to *Bradyrhizobium elkanii*, while in *recA* phylogeny they showed relatedness to *B*. *vignae*, an observation which could suggest recombination, migration or horizontal gene transfer [44, 46]. The DNA isolates with phylogenetically inconsistent behaviour were discrepant in their placement within the different housekeeping gene trees, and therefore grouped in different clusters. The discordant clustering of these isolates could be attributed to differences in the evolutionary history of the genes, horizontal gene transfer, and/or subsequent recombination events [47–48]. Many studies have shown that horizontal gene transfer plays a significant role in the evolution of rhizobia [49–50].

Recently, Gronemeyer *et al*. [45, 51–52] showed that *Bradyrhizobium subterraneum*, *Bradyrhizobium kavangense* and *Bradyrhizobium vignae* (all of African origin) are responsible for the nodulation of Bambara groundnut. However, from the phylogenetic analysis of individual and concatenated genes in this study, there were many unknown *Bradyrhizobium* species from Ghanaian and South African soils that nodulated Bambara groundnut, and are still waiting to be properly described. These include the nodule DNA isolates that formed their own separate clusters without any reference type strains in the phylogenetic trees shown in Figs 3–9. Clearly, the taxonomic position of microsymbionts nodulating Bambara groundnut is not yet well defined.

Nodulation (*nodD*) and N_2_-fixing (*nifH*) genes are major determinants of the outcome of any legume-*Rhizobium* symbiosis, and are therefore essential for nodulation and nitrogen fixation in legumes. However, these symbiotic genes *per se* are not responsible for the taxonomic position of rhizobia due to their location on interchangeable elements such as symbiotic islands and plasmids. Although the *nifH* gene phylogeny showed that nodule DNA isolates TUTVSBLG, TUTVSBEG, TUTVSRDM, TUTVSBEM, TUTVSBMK, TUTVSRMT, TUTVSCRM.I, TUTVSBMSA and TUTVSBEK in this study were associated with Bambara groundnut nodulation and could have a similar origin with *B*. *vignae*, the rest of the test samples were rather unique and novel.

Because of the regulatory function of *nod*D in the expression of other nodulation genes (e,g, *nod*ABC), this gene has been used as a symbiotic marker in the analysis of specificity between bacterial symbionts and their host plants. Although the *nodD* sequences of all test nodule DNA samples were not identical, they formed a separate clade on the *nodD* phylogeny. And except for TUTVSRMSA and TUTVSBMSA.I, all the nodule DNA isolates were proximally related to *Bradyrhizobium elkanii* USDA 3451 with 95.6 to 96.6% sequence identity. This reference strain USDA 3451 is the bacterial symbiont of *Macrotyloma africanum* and was isolated in Zimbabwe [53]. Bacterial DNA samples TUTVSRMSA and TUTVSBMSA.I also respectively showed a maximum of 92 and 91.3% sequence similarity with *B*. *elkanii* USDA 94, the microsymbiont of *Glycine max* [54].

Taken together, the findings of this study indicate that diverse native bradyrhizobia are responsible for the nodulation of Bambara groundnut in Ghana and South Africa. Detailed studies are however needed to unravel the true identity of the novel group of bradyrhizobia that did not cluster with any reference type strains in this report.

## Supporting information

S1 Table Temperature profiles and primer sets used in the PCR amplification. (DOCX)

S2 Table GenBank accession number of the gene sequences used in this study for Bambara groundnut nodulating rhizobial isolates. (DOCX)

S3 Table Nucleotide sequences of 16SrDNA, *nifH*, *nodD* and housekeeping genes used in phylogenetic analysis. (DOCX)
