# Supplementary material for: African origin of Bradyrhizobium populations nodulating Bambara groundnut (Vigna subterranea L. Verdc) in Ghanaian and South African soils
Source: PLoS One. 2017 Sep 25;12(9):e0184943. doi: 10.1371/journal.pone.0184943 (PMC5612659; doi:10.1371/journal.pone.0184943)
Supplement: S1 Table — (DOCX) [file pone.0184943.s001.docx]

**Table S1:** Temperature profiles and primer sets used in the PCR amplification

| **Target**  **gene** | **Temperature profile** | **Primer sequences** | **References** |
| --- | --- | --- | --- |
| 16SrDNA | 5 min 95 ̊C, 35 X (1 min 95 ̊C, 1 min 55 ̊C, 1 min 72 ̊C), 10 min 72 | F-5’AGAGTTTGATCCTGGCTCAG3’  R-5’CTTAAGGAGGTGATCCAGCC3’ | (1) |
| *nif*H | 5’ 94 ºC, 20x(0:30’94 ºC, 0:30’65 ºC, 0.5 per cycle, 1.30’72 ºC), 24x(0:30’94 ºC, 0:30’55 ºC, 1:30’72 ºC), 10’72 ºC | F: 5ʹTACGGNAARGGSGGNATCGGCAA3ʹ  R: 5ʹAGCATGTCYTCSAGYTCNTCCA3ʹ | (2) |
| *atp*D | 2’95 ºC, 34x(0.45’95 ºC, 0:30’65 ºC, 1:30’72 ºC), 10`72 ºC | F: 5'TCTGGTCCGYGGCCAGGAAG3'  R: 5'CGACACTTCCGARCCSGCCTG3' | (3) |
| *gln*II | 2’95 ºC, 34x(0.45’95 ºC, 0:30’65 ºC, 1:30’72 ºC), 10`72 ºC | F:5'AAGCTCGAGTACATCTGGCTCGACGG3'  R: 5'SGAGCCGTTCCAGTCGGTGTCG3' | (3) |
| *recA* | 2’95 ºC, 34x(0.45’98 ºC, 0.30’69.3 ºC, 1.30’72 ºC),10`72 ºC | F-5’CAACTGCMYTGCGTATCGTCGAAGG3’  R-5’CGGATCTGGTTGATGAAGATCACCATG3’ | (3) |
| *nodD* | 0.30’95 ºC, 40x(0.30’96 ºC, 1’53 ºC, 0:30’72 ºC), 05`72 ºC | F-5’GAT YGT CAT GAA ATC KGA GAG3’  R-5’TCG ATA GAA NAC ATC CAC ACG AT3’ | (4) |

1. Weisburg WG, Barns SM, Pelletier DA, Lane DJ. 16S ribossomal DNA amplification for phylogenetic study. J Bacteriol. 1991;173: 697-703.

2. Nzoué A, Miché L, Klonowska A, Laguerre G, de Lajudie P, Moulin L. Multilocus sequence analysis of bradyrhizobia isolated from Aeschynomene species in Senegal. Syst Appl Microbiol. 2009;32**:**400-412.

3. Stępkowski T, Żak M, Moulin L, Króliczak J, Golińska B, Narożna D, ... Mądrzak CJ. *Bradyrhizobium* *canariense* and *Bradyrhizobium* *japonicum* are the two dominant *Rhizobium* species in root nodules of lupin and serradella plants growing in Europe. Syst Appl Microbiol. 2011;34**:** 368-375.

4. Sterner JP, Parker MA. Diversity and relationships of bradyrhizobia from Amphicarpaea bracteata based on partial nod and ribosomal sequences. Syst Appl Microbiol. 1999;2(3):387-392.
