# Supplementary material for: African origin of Bradyrhizobium populations nodulating Bambara groundnut (Vigna subterranea L. Verdc) in Ghanaian and South African soils
Source: PLoS One. 2017 Sep 25;12(9):e0184943. doi: 10.1371/journal.pone.0184943 (PMC5612659; doi:10.1371/journal.pone.0184943)
Supplement: S2 Table — (DOCX) [file pone.0184943.s002.docx]

**Table S2.** GenBank accession number of the gene sequences used in this study for Bambara groundnut nodulating rhizobial isolates

|  | **16S rDNA** | ***atpD*** | ***glnII*** | ***recA*** | ***nifH*** | ***nodD*** |
| --- | --- | --- | --- | --- | --- | --- |
| TUTVSCRK |  | KT207514 | KT207526 |  | KT207550 |  |
| TUTVSRDK |  | KT207513 | KT207527 |  | KT207549 |  |
| TUTVSBLS | KT239677 | KT207509 | KT207531 |  |  | KY355124 |
| TUTVSBESA | KT239672 | KT207504 | KT207538 | KY355096 | KT207540 | KY355128 |
| TUTVSBLG |  | KT207519 | KT207520 | KT207558 | KT207556 | KY355114 |
| TUTVSBEG | KT239679 | KY355091 | KT207522 | KT207559 | KT207554 | KY355116 |
| TUTVSRDM | KT239678 | KT207517 | KT207523 | KT207560 | KT207553 | KY355117 |
| TUTVSRMK |  | KT207512 | KT207528 |  | KT207548 | KY355119 |
| TUTVSBEM | KY355089 | KT207516 | KT207524 | KT207561 | KT207552 |  |
| TUTVSRDS |  | KY355093 | KT207532 | KT207564 | KT207544 |  |
| TUTVSCRK-I | KT239674 | KY355095 | KT207536 |  |  | KY355126 |
| TUTVSRMSA | KT239673 | KT207505 | KT207537 | KY355098 | KT207541 | KY355127 |
| TUTVSRDM-I | KT239676 | KY355094 | KT207534 |  |  |  |
| TUTVSCRG |  | KT207518 | KT207521 |  | KT207555 | KY355115 |
| TUTVSBLK |  | KT207515 | KT207525 | KY355097 | KT207551 | KY355118 |
| TUTVSBEK |  |  | KT207529 | KT207562 | KT207547 | KY355120 |
| TUTVSRMT |  | KT207510 | KT207530 | KT207563 | KT207545 | KY355123 |
| TUTVSCRM-I | KY355090 | KT207508 | KT207533 |  | KT207543 | KY355125 |
| TUTVSBLK-I | KT239675 | KT207507 | KT207535 |  |  |  |
| TUTVSRDK-I |  | KT207506 |  |  | KT207542 |  |
| TUTVSBMK |  | KY355092 |  |  | KT207546 | KY355121 |
| TUTVSCRSA-I |  |  |  | KT207557 |  |  |
| TUTVSBMSA-I | KT239670 | KT207502 |  |  |  |  |
| TUTVSBMSA | KT239671 | KT207503 |  |  |  | KY355129 |
| TUTVSBLT |  | KT207511 |  |  |  | KY355122 |
