# Supplementary material for: African origin of Bradyrhizobium populations nodulating Bambara groundnut (Vigna subterranea L. Verdc) in Ghanaian and South African soils
Source: PLoS One. 2017 Sep 25;12(9):e0184943. doi: 10.1371/journal.pone.0184943 (PMC5612659; doi:10.1371/journal.pone.0184943)
Supplement: S3 Table — (DOCX) [file pone.0184943.s003.docx]

**Table S3** Nucleotide sequences of 16SrDNA, *nifH, nodD* and housekeeping genes used in phylogenetic analysis

| **Locus** | **No. of strains used for tree construction** | **Nucleotide sequence information** | | | | **Total*** | **Frequency**  **T/C/A/G**  **(%)** |
| --- | --- | --- | --- | --- | --- | --- | --- |
|  |  | **Cnoserved (C)** | **Variables (V)** | **Parsimony-informative (Pi)** | **Singleton (S)** |  |  |
| *16S rDNA* | 48 | 691 (63.16) | 367 (33.55) | 174 (15.90) | 189 (17.28) | 1094 | 20.6/24.2/24.1/31.1 |
| *atpD* | 50 | 243 (64.46) | 134 (35.54) | 81 (21.49) | 53 (14.06) | 377 | 15.9/33.0/18.7/32.4 |
| *gln*II | 55 | 199 (63.99) | 112 (36.01) | 89 (28.62) | 23 (7.4) | 311 | 20.5/29.3/16.1/34.1 |
| *recA* | 49 | 201 (53.32) | 136 (40.36) | 93 (27.60) | 43 (12.76) | 337 | 16.5/35.6/16.3/31.6 |
| Concatenated  (*glnII*+ *recA*) | 45 | 334 (61.85) | 206 (38.14) | 138 (25.55) | 68 (12.59) | 540 | 17.8/32.9/16.5/32.8 |
| (*glnII+recA+atpD*) | 33 | 488 (63.46) | 281 (36.54) | 178 (23.15) | 103 (13.39) | 769 | 17.0/33.3/17.0/32.6 |
| *nif*H | 53 | 154 (60.39) | 101 (39.61) | 85 (33.33) | 16 (6.27) | 255 | 20.0/27.4/19.2/33.4 |
| *nodD* | 28 | 109 (52.65) | 98 (47.34) | 68 (32.85) | 30 (14.49) | 207 | 19.0/29.8/18.7/32.5 |

*number of sites used for tree construction
